# Fisetin Regulates Nrf2 Expression and the Inflammation-Related Signaling Pathway to Prevent UVB-Induced Skin Damage in Hairless Mice

**DOI:** 10.3390/ijms18102118

**Published:** 2017-10-10

**Authors:** Po-Yuan Wu, Jia-Ling Lyu, Yi-Jung Liu, Ting-Yi Chien, Hao-Cheng Hsu, Kuo-Ching Wen, Hsiu-Mei Chiang

**Affiliations:** 1 Department of Dermatology, China Medical University Hospital, Taichung 404, Taiwan; wu.poyuan@gmail.com; 2 School of Medicine, China Medical University, Taichung 404, Taiwan; 3 Department of Cosmeceutics, China Medical University, Taichung 404, Taiwan; u105306601@cmu.edu.com (J.-L.L.); ella8175@gmail.com (Y.-J.L.); u105044701@cmu.edu.com (T.-Y.C.); ilj27@hotmail.com (H.-C.H.); kcwen0520@mail.cmu.edu.tw (K.-C.W.); 4 Ph.D Program for Biotechnology Industry, China Medical University, Taichung 404, Taiwan

**Keywords:** fisetin, photodamage, erythema, nuclear factor erythroid 2-related factor, filaggrin

## Abstract

Chronic ultraviolet (UV) exposure may cause skin damage, disrupt skin barrier function, and promote wrinkle formation. UV induces oxidative stress and inflammation, which results in extracellular matrix degradation in the dermis and epidermal hyperplasia. Our previous study demonstrated that fisetin exerts photoprotective activity by inhibiting mitogen-activated protein kinase/activator protein-1/matrix metalloproteinases (MMPs) activation. In this study, fisetin was applied topically to investigate its antiphotodamage effects in hairless mice. The erythema index (a* values) and transepidermal water loss were evaluated to assess skin damage, and immunohistochemical staining was conducted to elucidate the photoprotective mechanism of fisetin. The results revealed that the topical application of fisetin reduced UVB-induced increase in the a* value and wrinkle formation. In addition, fisetin inhibited epidermal hyperplasia and increased the collagen content in the dermis. Fisetin exerted photoprotective activity by inhibiting the expression of MMP-1, MMP-2, and cyclooxygenase-2 and increasing the expression of nuclear factor erythroid 2-related factor. Furthermore, fisetin increased the expression of filaggrin to prevent UVB-induced barrier function disruption. Altogether, the present results provide evidence of the effects and mechanisms of fisetin’s antiphotodamage and antiphotoinflammation activities.

## 1. Introduction

The skin is an external organ that acts as a crucial barrier and defense system to prevent water loss and damage due to sunlight exposure and environmental pollution. Ultraviolet (UV) irradiation induces reactive oxygen species (ROS) generation and inflammation in the dermis [1,2]. Long-term UV exposure causes skin sagging or wrinkle formation because of the degradation of extracellular matrix (ECM) proteins, including elastin, collagen, and fibronectin [3]. Collagen is the main structural protein in the dermis and is synthesized in fibroblasts. UV irradiation activates the signaling transduction pathway related to collagen degradation, causing the aforementioned fragmentation of collagen fibers that leads to skin sagging and wrinkle formation. Collagen synthesis is regulated by upstream factors such as the activity of mitogen-activated protein kinase (MAPK), activator protein-1 (AP-1), and matrix metalloproteinases (MMPs). MMPs play vital roles in skin aging and photoaging processes. UV-induced MMP activation triggers ECM ubiquitination, which causes skin damage and wrinkle formation [4,5].

UV irradiation may cause oxidative stress by ROS generation. ROS interact with cellular biomolecules such as proteins, lipids, and nucleic acids and alters the redox status in skin cells [6]. In addition, antioxidant-response element (ARE) is critical for the regulation of many genes that encode antioxidant proteins, such as heme oxygenase-1 (HO-1) [7]. Nuclear factor erythroid 2-related factor (Nrf2) binds to ARE to regulate the transcriptional activation of antioxidant genes. Nrf2/ARE/HO-1 regulation neutralizes oxidative stress [7]. UV irradiation induces oxidative stress and inhibits the expression and activity of Nrf2, thus causing skin damage.

In addition to ECM damage, UV overexposure can result in the generation of proinflammatory mediators, leading to skin inflammation. Interleukins (ILs) and nitric oxide (NO) play crucial roles in UV-induced skin inflammatory processes [8]. An increase in NO synthase (NOS) and cyclooxygenase (COX) causes skin erythema and inflammation. UVB irradiation induces skin erythema, which is related to vascular dilation. UV exposure upregulates inducible NOS (iNOS) expression and subsequently promotes NO generation, which stimulates peroxynitrite and ROS generation and upregulates COX-2 expression [9]. Nuclear factor kappa B (NF-κB) is a transcription factor that modulates proinflammatory cytokines such as tumor necrosis factor-alpha (TNF-α) and ILs, resulting in hazardous effects [10,11]. UV exposure causes NF-κB translocation into the nucleus and triggers the inflammation and degradation of the ECM in the dermis [10,12].

Fisetin is a flavonoid that is abundant in fruits and vegetables [13]. It exhibits chemoprevention, antioxidant, and anti-inflammatory activities [14,15,16,17]. A previous study reported that fisetin increases the survival rate of retinal pigment epithelial cells through p53 acetylation and decreased SIRT1 levels [15]. In addition, fisetin inhibited TNF-α-induced inflammation and hydrogen peroxide-induced oxidative damage in human keratinocytes [18]. It also inhibited the MAPK and NF-κB signaling cascade in BRAF-mutated melanoma cells to block their invasion ability. In addition, fisetin inhibited the proliferation of melanoma cells [19,20], as well as ultraviolet B (UVB)-induced phosphoinositide 3-kinase (PI3K) expression, protein kinase B (PKB; AKT) phosphorylation, and NF-κB activation in the skin of SKH-1 mice [19]. Our previous study demonstrated that fisetin alleviates UVB-induced MAPK phosphorylation and elevated MMP expression and inflammation responses in human skin fibroblasts [21]. The present study further investigated the effects and mechanisms of fisetin’s antiphotodamage activity by modulating the Nrf-2 signaling pathway in hairless mice exposed to UVB irradiation. In addition, the expression of the collagen degradation protein and inflammatory-related protein and skin barrier function were examined.

## 2. Results

### 2.1. Fisetin Protects Hairless Mice from UVB-Induced Skin Damage

In this study, female BALB/c hairless mice were exposed to UVB irradiation, and fisetin was applied topically for 10 weeks. At the end of the experiment, no considerable differences were observed in the body weights of the mice in all five groups.

**Figure 1:**
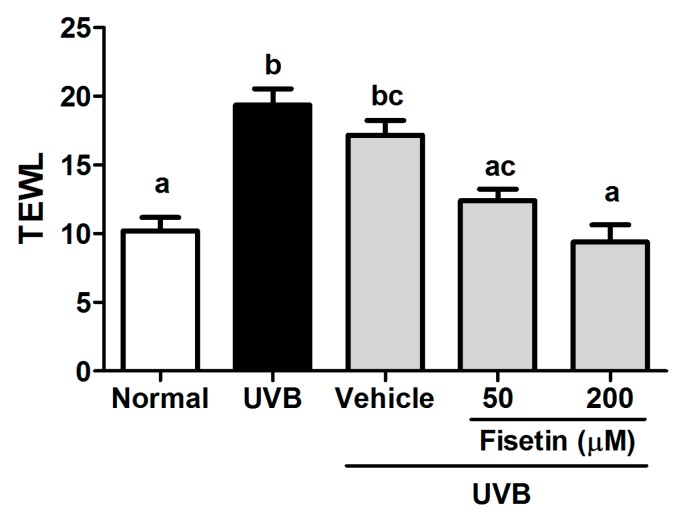
Effect of fisetin on transepidermal water loss (TEWL) in the ultraviolet B (UVB)-irradiated hairless mice at the 10th week. a–c: Values not followed by a common letter are significantly different (*p* < 0.05).

Transepidermal water loss (TEWL) is a marker for skin barrier function. TEWL increases when the stratum corneum is disrupted by physical factors or chemicals. The present results indicated that UVB exposure considerably enhances TEWL in mice. The topical application of fisetin alleviated TEWL induced by UVB exposure (Figure 1), indicating that fisetin maintained skin barrier function during UVB exposure.

### 2.2. Fisetin Protects Hairless Mice from UVB-Induced Skin Inflammation

**Figure 2:**
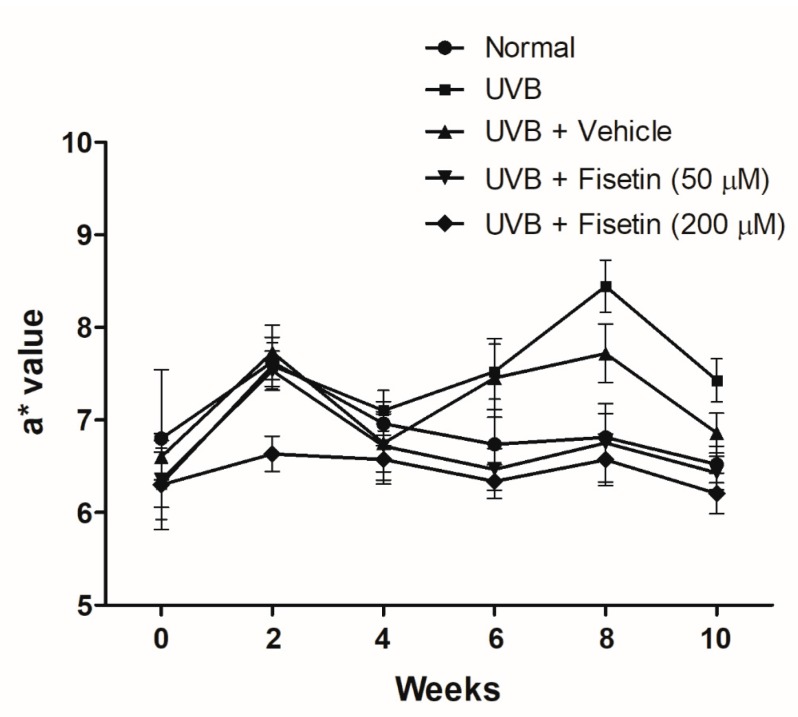
Effects of fisetin on the a* values in the UVB-irradiated hairless mice.

The a* value is an index of erythema that represents the degree of skin inflammation. UVB exposure resulted in elevated a* values, indicating that UVB exposure causes skin inflammation and erythema (Figure 2). However, fisetin treatment reduced the a* values measured in the 6th–10th weeks. These results suggested that fisetin reduces UVB-induced skin inflammation.

### 2.3. Effects of Fisetin on UVB-Induced Skin Aging

**Figure 3:**
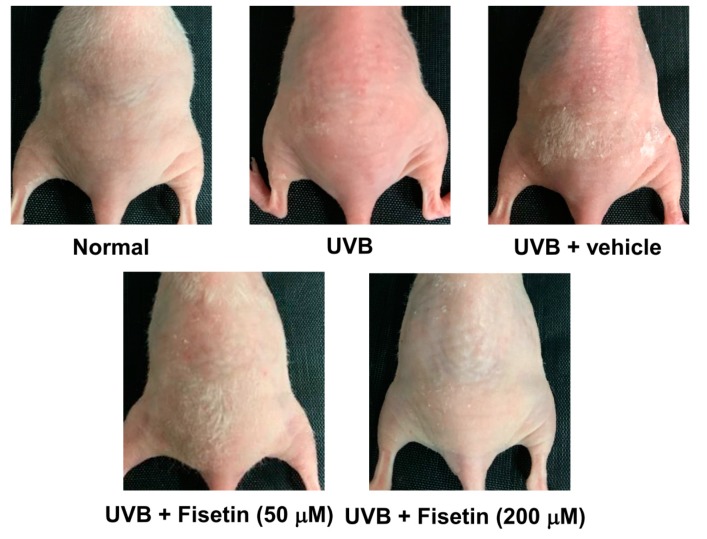
Images of UVB-induced skin wrinkles and the effects of topically applied fisetin.

Wrinkles were observed on the dorsal region in the UVB-irradiated mice (Figure 3). However, the topical application of fisetin reduced wrinkle formation. The wrinkle scores of UVB-irradiated and 50 μM fisetin-treated groups were 4.6 ± 1.5 and 2.7 ± 1.5, respectively (Table 1). Fisetin treatment significantly reduced wrinkles on the dorsal skin of the mice.

**Figure 4:**
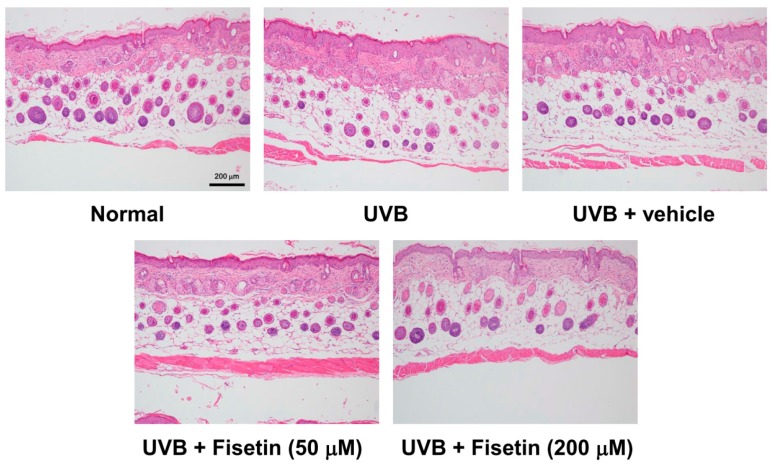
Light micrographs of the histological sections stained with hematoxylin and eosin in the hairless mice.

**Figure 5:**
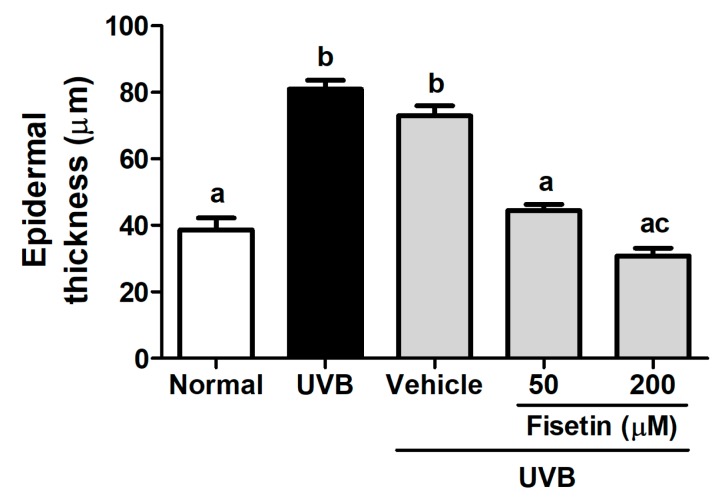
Effects of fisetin on the epidermal thickness of the UVB-irradiated hairless mice in the 10th week. a–c: Values not followed by a common letter are significantly different (*p* < 0.05).

**Figure 6:**
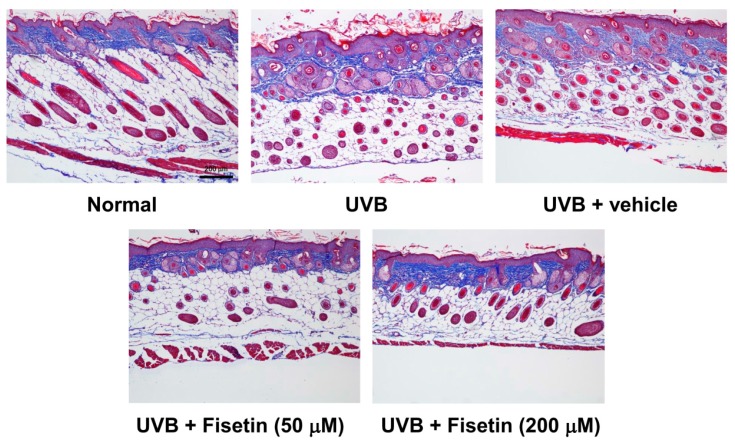
Light micrographs of the histological sections of the hairless mice stained with Masson trichrome. Collagen fibers are stained in blue in the dermis.

To further understand the effects of fisetin on UVB-induced skin aging, histological examinations were performed to determine the epidermal thickness and collagen content of the skin. The epidermal thickness was 38.61 ± 11.39 μm in the control group, which increased to 80.98 ± 8.34 μm in the UVB-irradiated group (Figure 4 and Figure 5). However, fisetin treatment (50 and 200 μM) significantly reduced the epidermal thickness induced by UVB-irradiated group. The epidermal thickness decreased by 62% in the UVB-irradiated 200 μM fisetin-treated group compared with the UVB-irradiated group. The results indicated that the topical application of fisetin ameliorates UVB-induced skin hyperplasia. In addition, Masson trichrome staining results revealed that the collagen content in the dorsal skin decreased considerably after UVB exposure. The topical application of fisetin on the dorsal skin for 10 weeks increased the collagen content in the dermis (Figure 6).

### 2.4. Fisetin Reduces UVB-Induced Collagen Degradation by Inhibiting MMP-1 and MMP-2 Expression in the Dorsal Skin of Hairless Mice

**Figure 7:**
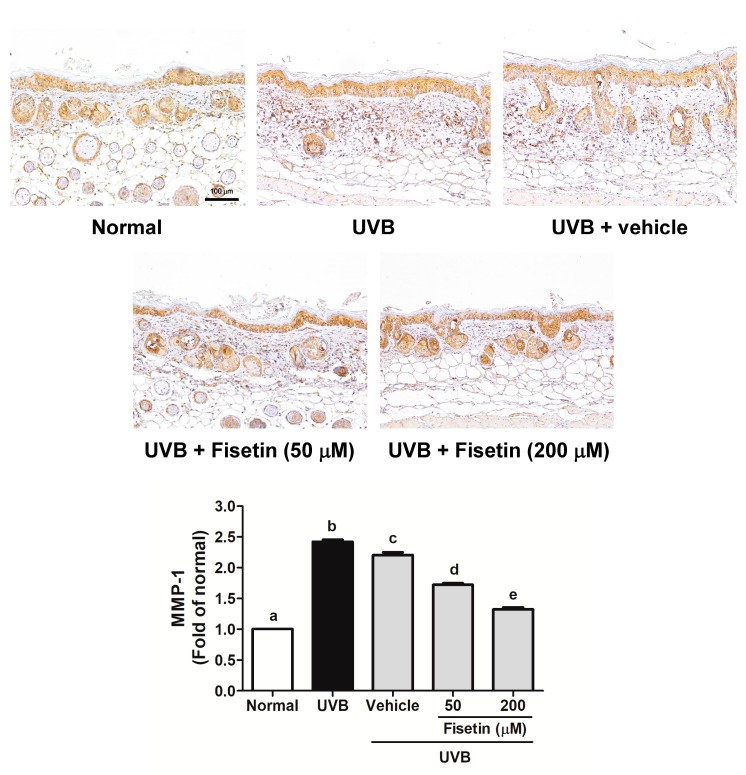
Immunohistochemical staining of MMP-1 expression in the dermis of the mouse skin slices. Fisetin inhibits UVB-induced MMP-1 overexpression (200×). a–e: Values not followed by a common letter are significantly different (*p* < 0.05).

**Figure 8:**
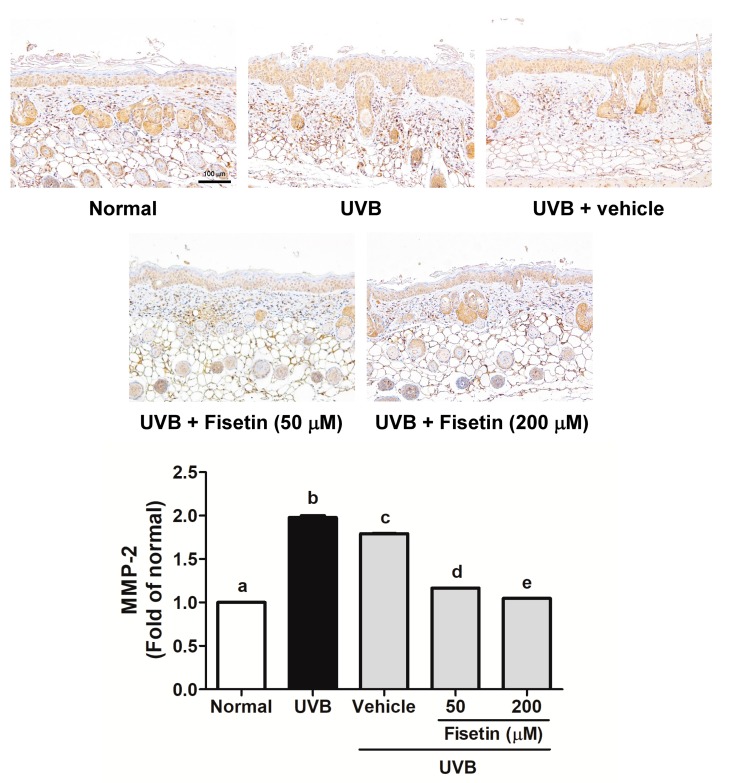
Immunohistochemical staining of MMP-2 expression in the dermis of the mouse skin slices. Fisetin inhibits UVB-induced MMP-2 overexpression. a–e: Values not followed by a common letter are significantly different (*p* < 0.05).

UVB-induced MMP-1 and MMP-2 overexpression induced collagen degradation in the skin dermis (Figure 7 and Figure 8). MMP-1 expression increased after UVB exposure, whereas fisetin treatment reduced MMP-1 expression (Figure 7). It was interesting that topical application of vehicle (polyethylene glycol (PEG) 400) would decrease MMP-1 expression. Similar results were obtained with regard to MMP-2 expression (Figure 8).

### 2.5. Fisetin Reduces UVB-Induced Skin Inflammation by Inhibiting the Expression of COX-2, IL-6, and NF-κB in the Dorsal Skin of Hairless Mice

**Figure 9:**
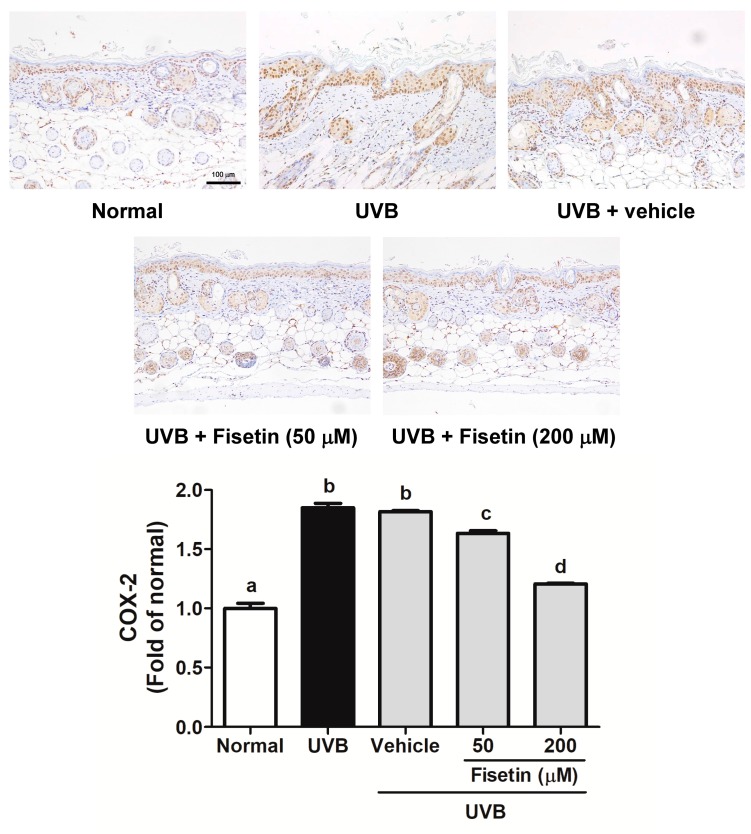
Immunohistochemical staining of COX-2 expression in the epidermis of the mouse skin slices. Fisetin inhibits UVB-induced COX-2 overexpression (200×). a–d: Values not followed by a common letter are significantly different (*p* < 0.05).

**Figure 10:**
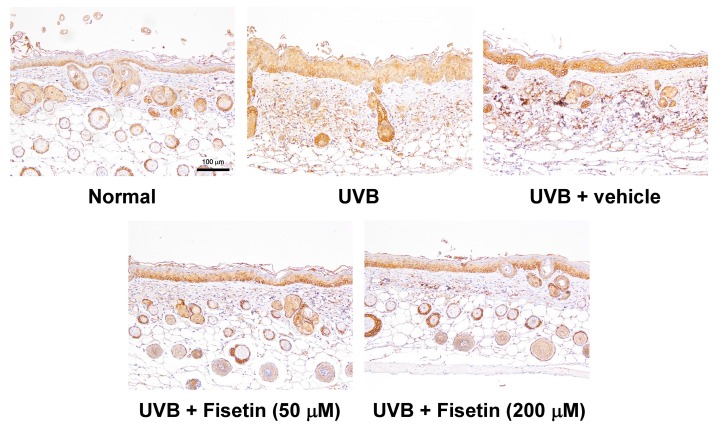

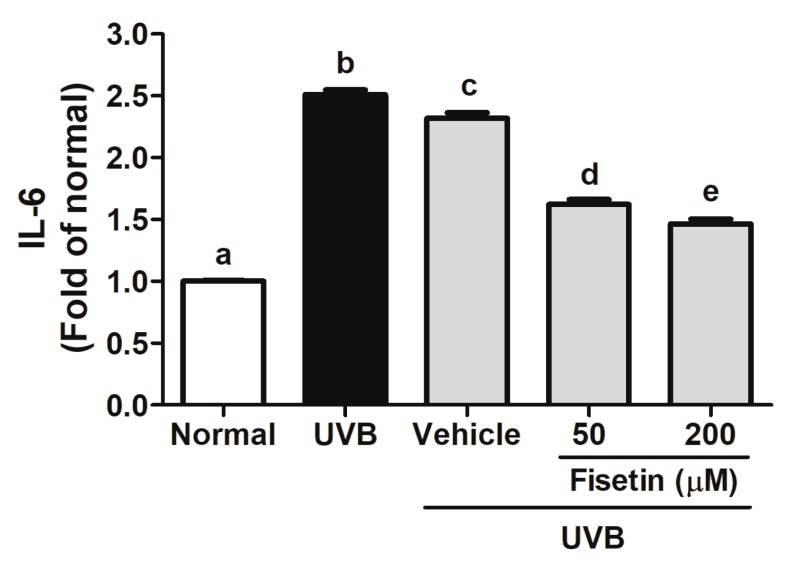
Immunohistochemical staining of IL-6 expression in the epidermis of the mouse skin slices. Fisetin inhibits UVB-induced IL-6 overexpression (200×). a–e: Values not followed by a common letter are significantly different (*p* < 0.05).

**Figure 11:**
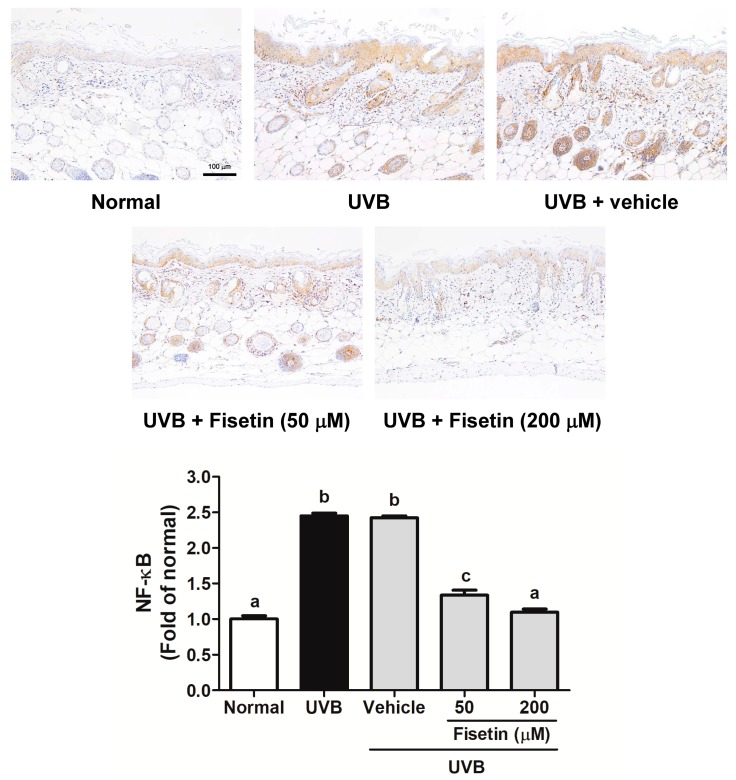
Immunohistochemical staining of NF-κB expression in the epidermis of the mouse skin slices. Fisetin inhibits UVB-induced NF-κB overexpression (200×). a–c: Values not followed by a common letter are significantly different (*p* < 0.05).

UVB upregulates inflammatory cytokines and related factors, causing skin damage. Figure 9 shows that UVB induces COX-2 overexpression in mouse skin, whereas the topical application of fisetin significantly inhibits UVB-induced inflammation. Similar results were obtained with regard to UVB-induced IL-6 and NF-κB upregulation, which was inhibited after fisetin treatment (Figure 10 and Figure 11). In addition, the effects of a high fisetin dose (200 μM) were significantly superior to those of a low fisetin dose (50 μM).

### 2.6. Fisetin Reduces UVB-Induced Oxidative Stress by Increasing Nrf2 Expression in the Dorsal Skin of Hairless Mice

**Figure 12:**
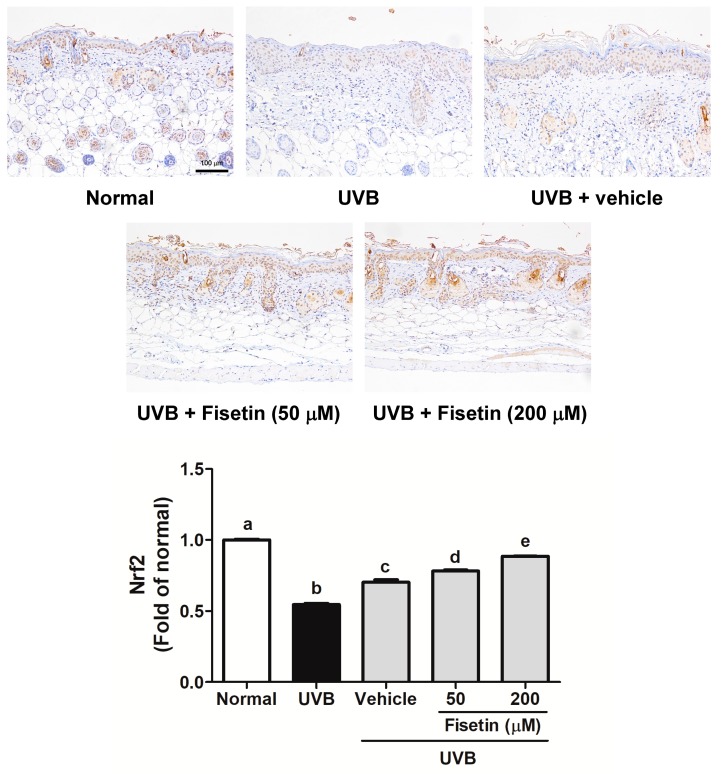
Immunohistochemical staining of Nrf2 expression in the epidermis of the mouse skin slices. Fisetin increases Nrf2 overexpression (200×). a–e: Values not followed by a common letter are significantly different (*p* < 0.05).

UVB triggers ROS generation in the skin to disrupt skin barrier functioning and cause damage, and Nrf2 to neutralize oxidative stress. Figure 12 shows that UVB irradiation inhibited Nrf2 expression in the mouse skin, and the topical application of fisetin significant upregulated Nrf2 expression.

### 2.7. Fisetin Reduces the UVB-Induced Disruption of Skin Barrier Function by Increasing Aquaporin and Filaggrin Expression in the Dorsal Skin of Hairless Mice

**Figure 13:**
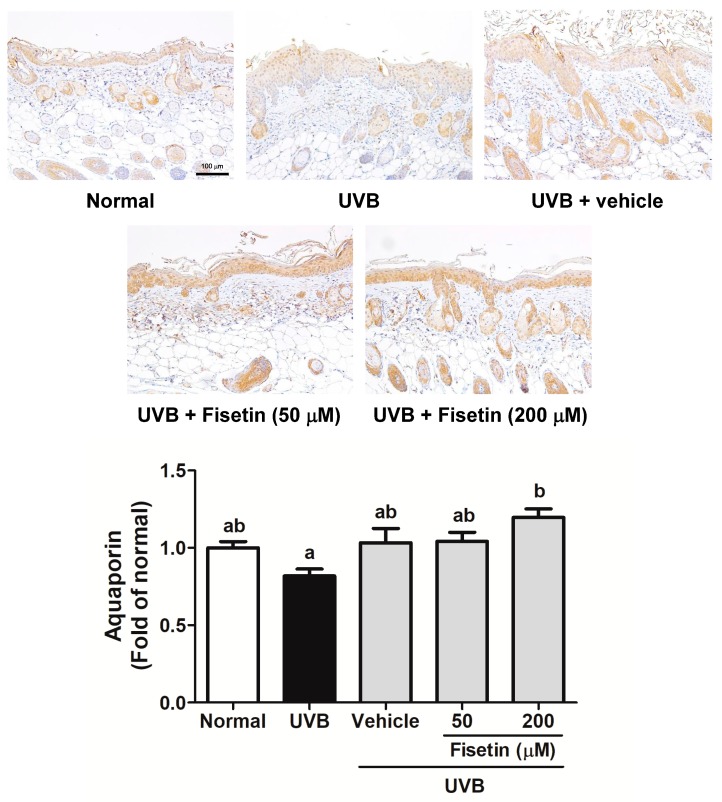
Immunohistochemical staining of aquaporin expression in the epidermis of the mouse skin slices. Fisetin increases aquaporin overexpression (200×). a,b: Values not followed by a common letter are significantly different (*p* < 0.05).

**Figure 14:**
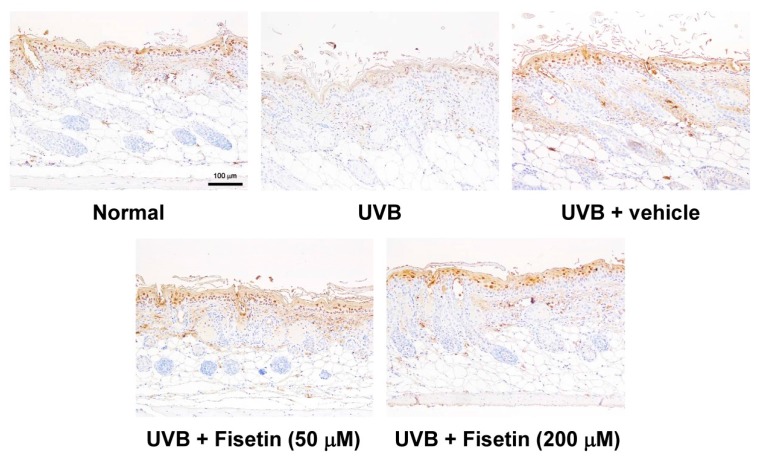

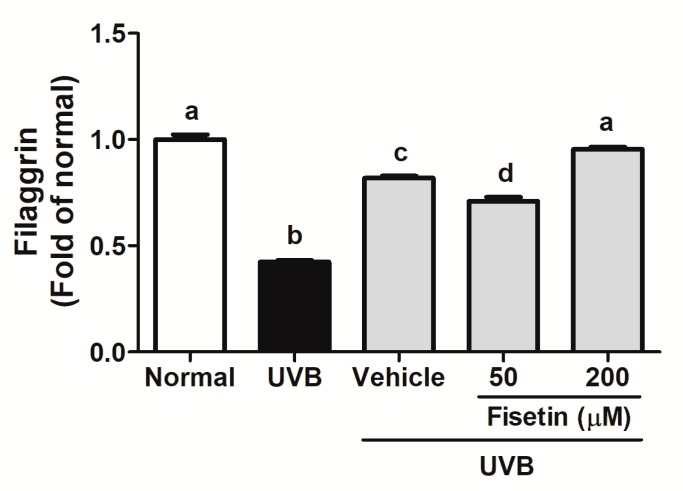
Immunohistochemical staining of filaggrin expression in the epidermis of mouse skin slices. Fisetin increases filaggrin overexpression (200×). a–d: Values not followed by a common letter are significantly different (*p* < 0.05).

Chronic UVB exposure downregulated aquaporin and filaggrin expression in epidermis of the mouse skin (Figure 13 and Figure 14). However, the topical application of fisetin significantly increased filaggrin expression. Fisetin also significantly increased aquaporin expression at high dose. It was interesting that topical application of vehicle would increase filaggrin expression, but not aquaporin. In addition, 200 μM fisetin treatment significantly increased aquaporin expression compared with 50 μM fisetin treatment.

## 3. Discussion

UV irradiation enhances ROS formation to disturb the balance between the synthesis and degradation of collagen. It also upregulates MMPs to promote ECM degradation and inhibits collagen production in the dermis, resulting in skin photoaging [1,22,23]. The topical application of antioxidants and anti-inflammatory agents may ameliorate UV-induced photodamage [23,24]. A previous study demonstrated that fisetin protects the skin from UVB-induced damage by inhibiting ROS generation and the MAPK/AP-1/MMP signaling pathway, decreasing collagen degradation in fibroblasts [21]. In the present study, the topical application of fisetin for 10 weeks reduced wrinkle formation and collagen degradation in the mouse dorsal skin, thus protecting against photodamage. Studies have reported that fisetin exhibits antioxidant and anti-inflammatory activities, which may contribute to its antiphotodamage effects [14,15,16,17]. In addition, fisetin inhibited UVB-induced MMP overexpression to ameliorate collagen degradation in the dermis.

UVB exposure may increase the expression levels of proinflammatory factors, such as COX-2 and ILs [9,22]. IL-6 is associated with various cutaneous inflammatory disorders, such as atopic dermatitis, psoriasis, and photoaging [25]. The present results demonstrate that fisetin reduced UVB-induced IL-6 production. A previous study reported that fisetin inhibited COX-2 overexpression and inhibitor κB degradation, increased NF-κB levels in the cytoplasm, and reduced NF-κB translocation into the nucleus of fibroblasts. Furthermore, fisetin inhibited UV-induced production of prostaglandin E2 and NO in human skin fibroblasts [21]. In the present study, fisetin inhibited UVB-induced COX-2, iNOS, and NF-κB overexpression, protecting the dorsal skin of the hairless mice from photoinflammation. Moreover, the upregulation of ILs and NF-κB may activate COX-2 expression in aged skin fibroblasts and keratinocytes [26]. Our previous study revealed that fisetin treatment inhibits MAPK phosphorylation, which may inhibit NF-κB activation. NF-κB activates the expression of genes involved in inflammatory and immunological responses [27]. In addition, the translocation of NF-κB into the nucleus induces the transcription of proinflammatory cytokines, including iNOS, ILs, and COX-2, which were inhibited by fisetin treatment. The present results are consistent with those of the previous study; therefore, fisetin inhibited MMP, COX-2, and NF-κB expression in fibroblasts and the mouse skin. UV irradiation induces the translocation of NF-κB into the nucleus and elevates MMP expression, leading to collagen degradation in human skin.

Filaggrin is a structural protein in the stratum corneum that plays a vital role in the skin barrier function [28]. Free filaggrin binds with keratin intermediate filaments, which aggregate into macrofibrils to form a tightly packed structure expressed during terminal keratinocyte differentiation [29]. At the outer layers of the stratum corneum, filaggrin is degraded into free amino acids, collectively referred to as natural moisturizing factors, which contribute to epidermal hydration and barrier function [30]. Filaggrin deficiency results in the disruption of skin barrier function, and chronic UV exposure downregulates filaggrin [31].

Aquaporins (AQPs) are integral epidermal cell membrane proteins that facilitate water flow in and out of the cell. They are involved in various cellular functions, such as cell proliferation and migration. AQP3 expression is involved in skin diseases, including wound healing, atopic dermatitis, and psoriasis, the pathogenesis of which is attributed to AQP3’s water or glycerol transport function in epidermal keratinocytes [32]. In addition, chronic UV exposure damages skin integrity and increases water loss through the skin, causing dryness. The results of this study suggested that topical application of vehicle (PEG400) for 10 weeks may decrease the expression of MMP-1, -2, and IL-6, while increasing the expression of filaggrin and aquaporin. Polyols act as a humectant to increase the hydration of the skin and improve the barrier function; therefore, they are used as fundamental skin care [33,34]. It was reported that local application of glycerol and xylitol increased skin hydration and protein expression of filaggrin [35]. In addition, the results indicated that effects of fisetin on skin protection were superior to that of PEG. Our results indicate that the topical application of fisetin reduces TEWL and increases the filaggrin content in the stratum corneum. In addition, fisetin enhances aquaporin content in the skin. Altogether, fisetin ameliorates UVB-induced skin damage by inhibiting aquaporin and filaggrin damage, thus reducing water loss and preventing skin drying.

## 4. Materials and Methods 

### 4.1. Materials

Fisetin, DL-dithiothreitol, phenylmethylsulfonyl fluoride, and Triton X-100 were purchased from Sigma-Aldrich Chemical Corporation (St. Louis, MO, USA). MMP-1, MMP-2, COX-2, and Nrf2 antibodies were purchased from Genetex (Beverly, MA, USA). All reagents and chemicals used for the experiments were analysis grade.

### 4.2. Experimental Animals

All experimental protocols were approved by the institutional animal use and care committee of China Medical University (Protocol No.: 104-240-B, 19/2/2015). Five-week-old female BALB/cAnN.Cg-Foxn1nu/CrlNarl hairless mice were obtained from the National Laboratory Animal Center (Taipei, Taiwan). The mice were maintained under controlled conditions: temperature, 22 °C ± 2 °C; relative humidity, 50 ± 10%; light–dark cycle: 12 h.

### 4.3. UVB Irradiation and Fisetin Treatment

After the 1-week acclimatization, the mice were divided into five groups: control, UVB-irradiated, vehicle (polyethylene glycol (PEG) 400)-treated and UVB-irradiated, UVB-irradiated and 25 μM fisetin-treated, and UVB-irradiated and 100 μM fisetin-treated. Each group had five mice. A UV light (a broadband with a peak emission at 302 nm, CL-1000 M, UVP, USA) was used. The mice were exposed to a gradually increased dose of UVB irradiation three times a week: first week, 36 mJ/cm^2^; second–fourth weeks, 54 mJ/cm^2^; fifth–seventh weeks, 72 mJ/cm^2^; and 8th–10th weeks, 108 mJ/cm^2^, as described previously [36,37]. The vehicle-treated UVB-irradiated mice were topically administered 50 μL of PEG daily, and the fisetin-treated mice were topically administered 50 μL of 25 or 100 μM fisetin prepared in PEG 400 daily. Fisetin was applied on the skin after UVB irradiation. The control mice did not receive any treatment. All treatments were administered to the dorsal skin.

### 4.4. Measurement of Wrinkle Formation on the Dorsal Skin

The wrinkle formation on the dorsal skin of mice was observed macroscopically. The degree of wrinkle formation was evaluated from the photograph of each mouse according to the grading scale described previously, while the name of the group was unrevealed to the researcher [36].

### 4.5. Measurement of Skin Erythema and TEWL

To evaluate the effects of fisetin on UVB-induced erythema, erythema was tested every 2 weeks by using a spectrocolorimeter (SCM-104/108, Ruyico Technology Corporation, Taipei, Taiwan), as described previously [37,38]. The erythema was showed as a* value, which represents the color opponents green–red and is present as green at negative a* values and red at positive a* values. In addition, TEWL from the dorsal skin was measured at the 10th week with a Tewameter TM 300 (Courage + Khazaka Electronic GmbH, Cologne, Germany).

### 4.6. Preparation of Skin Specimens and Hematoxylin and Eosin Staining

The mice were sacrificed, and the dorsal skin samples were excised and fixed in 10% formaldehyde and embedded in paraffin as described previously [37,38]. The skin slides were stained in hematoxylin and eosin, and the skin thickness of each section was determined using Image J software, as described previously (National Institutes of Health, Bethesda, MD, USA). Collagen in the skin sections was stained using Masson trichrome and examined under a microscope [38].

### 4.7. Immunohistochemical Staining

The skin sections were stained with the following monoclonal antimouse antibodies: MMP-1, MMP-2, IL-6, COX-2, NF-κB, Nrf-2, aquaporin, and filaggrin (Genetex, Beverly, MA, USA). The skin sections were examined under a microscope, and the protein expression was determined using Image J software, as described previously (National Institutes of Health, Bethesda, MD, USA) [38].

### 4.8. Statistical Analysis

The results were expressed as means ± standard deviations. In the quantitation of immunohistochemical staining, two stained slides for each animal were measured. Significant differences between groups in the experiments were analyzed using analysis of variance with least significant difference post hoc tests. A *p* value of <0.05 was considered statistically significant.

## 5. Conclusions

Our results demonstrate that fisetin attenuates UVB-induced damage, oxidative stress, inflammation, and skin barrier function disruption. Fisetin inhibits the UVB-induced expression of MMPs, COX-2, IL-6, and NF-κB, thus protecting the skin from wrinkles and erythema. Fisetin also increases the expression of Nrf2 in the mice skin. In addition, fisetin effectively inhibits UV-induced aquaporin and filaggrin damage. The present study demonstrates the protective effects of fisetin against UV-induced skin damage.

## Figures and Tables

**Table 1:** Effects of fisetin on UVB-induced skin wrinkles in the hairless mice.

Group	Wrinkle Score (10 Weeks)
Normal mice	1.6 ± 1.5 ^a^
UVB-irradiated mice	4.6 ± 1.5 ^b^
UVB-irradiated mice + vehicle	3.8 ± 1.4 ^b^
UVB-irradiated mice + Fisetin (50 μM)	2.7 ± 1.5 ^a^
UVB-irradiated mice + Fisetin (200 μM)	2.5 ± 1.8 ^ac^

**^a^**^–c^: Values not followed by a common letter are significantly different (*p* < 0.05).

## Abbreviations

UV	Ultraviolet
TEWL	Transepidermal Water Loss
CREB	Cyclic AMP Response Element-Binding Protein
MAP kinase	Mitogen-Activated Protein Kinase
AP-1	Activator Protein-1
ECM	Extracellular Matrix
MMP	Matrix Metalloproteinase
NO	Nitric Oxide
iNOS	Inducible Nitrite Oxide Synthase
COX-2	Cyclooxygenase-2
IκB	Inhibitor Kappa B
NF-κB	Nuclear Factor Kappa B
Nrf-2	Nuclear Factor Erythroid 2-Related Factor
ARE	Antioxidant-Response Element
HO-1	Heme Oxygenase-1
